# Revisiting the Organismic Valuing Process Theory of Personal Growth: A Theoretical Review of Rogers and Its Connection to Positive Psychology

**DOI:** 10.3389/fpsyg.2020.01706

**Published:** 2020-07-21

**Authors:** Mia M. Maurer, Daiva Daukantaitė

**Affiliations:** Department of Psychology, Lund University, Lund, Sweden

**Keywords:** organismic valuing process, personal growth, integration, humanistic psychology, positive psychology, well-being

## Abstract

Humanistic and positive psychology have had a contentious past. Initially, positive psychology researchers have distanced themselves from humanistic psychology, proceeding to build an array of differentiated constructs relevant to an empirical study of well-being. Twenty years on, it is now generally acknowledged that humanistic psychology is the theoretical predecessor of positive psychology in terms of holistic growth theories. In this theoretical review, we aim to show how Carl Rogers’ organismic valuing process (OVP) theory can serve as a holistic framework for individual positive psychological research findings and theories and how positive psychology, in turn, provides empirical support for this meta-theoretical framework. An important motivation for considering personal growth as a process that integrates various aspects of well-being is theoretical integration, which can help us better understand how well-being develops in individuals across the lifespan. Some theoretical and practical implications of incorporating OVP theory into well-being research are also suggested.

## Introduction

Positive psychological research on well-being has been accumulating since the field’s inception in the 1990s, which has considerably enhanced our understanding of the concept of “flourishing” and the constituents of the “good life.” Several decades previously, in the 1950s, humanistic psychology provided pioneering research and theory on the positive aspects of human life. Both of these fields have aimed to further our knowledge and understanding of the conditions of the good life, human well-being, and positive institutions. However, despite their similarity in aims, some researchers (particularly those in positive psychology) perceive them to have conflicting philosophical foundations (e.g., Waterman, 2013). As suggested by Friedman (2008), the biggest conflict between humanistic and positive psychology is their differing epistemologies and methodologies. Positive psychology is based on a logical positivist epistemology, emphasizing reductionism and quantitative research methods, whereas humanistic psychology is based on a holistic (subjectivist) epistemology, is antireductionist, and uses mainly qualitative methods (Friedman, 2008). Initially, Seligman and Csikszentmihalyi (2000) suggested that humanistic psychology lacked the empirical research basis of positive psychology. A more exhaustive account of this divide is beyond the scope of this article but has been discussed elsewhere (see Friedman, 2008; Waterman, 2013). However, some researchers have recognized that the two approaches have more similarities than differences (e.g., Joseph, 2015; Proctor et al., 2015) and that integrating these fields can only benefit them (e.g., Wong, 2011b; Kaufman, 2020). In line with these favorable developments, in this article, we seek to show how a humanistic meta-theoretical perspective on well-being as a process—namely, Carl Rogers’ (1959, 1964) organismic valuing process (OVP)—can serve as a holistic framework for individual positive psychological research findings and theories. Furthermore, we aim to show how positive psychology (along with some other subfields, such as attachment theory) provides empirical support for this meta-theoretical framework. In this way, both fields can support each other and draw from each other’s strengths.

What, however, would motivate us to revisit the OVP theory for growth toward well-being? One major motivation is theoretical integration, which would help us better understand how well-being develops in individuals across life. The case for theoretical integration was also made by Magnusson and Törestad (1993) in their description of how the progression of scientific understanding follows two main stages: (1) differentiation of knowledge, or building an ever greater and more nuanced understanding of individual concepts and constructs in an area of inquiry (in their case, the area was personality research), and (2) integration of this nuanced knowledge into more holistic theories whereby the ways in which the individual components are interrelated can be better understood as a holistic, unfolding process of growth. While positive psychology has been doing an impressive job with regard to the first stage, so far, the second stage has received little attention. By revisiting OVP theory, it might be possible to integrate our knowledge on different aspects of well-being to get a holistic picture of individual growth across life.

First, we will describe the main assertions of OVP theory and its components. Second, we will discuss recent positive psychological evidence in support each of these assertions. Finally, we will describe possible implications of incorporating the OVP theory into well-being research, particularly in applied contexts where well-being and growth are being promoted.

## Part 1: Main Assertions of the Organismic Valuing Process Theory of Growth

The OVP is a holistic growth theory that encompasses motivation-based theories of well-being (i.e., goal pursuits and self-determination), explains how the emotional and cognitive process of growth unfolds as an embodied “moment of movement,” and describes how this process is crucially affected by socioenvironmental conditions as well as changes in one’s way of relating to the outside world (Rogers, 1959, 1961, 1964).

As discussed by Rogers (1961, 1964) and summarized by Proctor et al. (2015), the OVP theory captures the way in which an individual gains a different way of relating both to the self and outer social world, and how the social context can nurture or stifle this process. In the following passages, the OVP theory of growth is briefly summarized by discussing its seven main assertions.

### The Actualizing Tendency

According to the OVP theory, all organisms (individuals) are naturally motivated toward growth, both physically and psychologically (Rogers, 1961, 1964). Such psychological growth, however, does not merely encompass learning cognitive skills but also growth toward greater well-being (becoming “fully functioning” in Rogers’ terms).

The actualizing tendency refers to an active push for the organism to improve itself via better organization, fulfillment of its potential, and growth. Psychologically, the actualizing tendency manifests as greater personal growth—initially, as a greater understanding and connection with the self (Rogers, 1959, 1961, 1964). Gradually, the individual learns to be open toward their own reactions within a given situation and be capable of openly observing and understanding how they react and feel. This transparency of emotions and thoughts might at first be frightening, but over time, the individual learns to accept the fluctuating reactions and can recognize the underlying assumptions that they have created in order to construe different situations in their lives (Rogers, 1961). They, in other words, learn to communicate with themselves and gain insight into their own “phenomenal field” (i.e., internal working models). This connection with the self can gradually become the primary force when support has been established; that is, one learns unconditional positive self-regard. Furthermore, a place of greater inner security can release greater compassion toward others. This entire actualizing process is not fast and requires great openness and self-awareness (Rogers, 1961).

### Conditions for Growth: A Psychologically Safe Atmosphere of Unconditional Positive Regard, Empathy, and Congruence

In order to elicit the actualizing tendency, the qualities of the psychosocial environment play a crucial role. Both the growth process and the organismic valuing process require favorable conditions (Rogers, 1961) particularly the esteem of another person without conditions of worth (i.e., imposing external demands and pressures for one to be accepted). Conditions of worth are viewed as the source of psychopathology (Rogers, 1959, 1961). They refer to external demands of one’s worthiness, or the idea that one is only worthy of love and belonging once one has fulfilled certain demands.

Rogers (1959) named the necessary and sufficient conditions for a person to start engaging with their organismic growth process: first, congruence, or genuineness—the growth-facilitating atmosphere is one in which there is a transparency of experience and expression, genuine presence. The second condition is unconditional positive regard, or the sense of appreciating the other as the whole of who they are—appreciation for one’s intrinsic worth. The third condition is empathic understanding, namely, the ability to reflect someone else’s inner experience, to attentively listen and see the world from that other’s frame of reference, and to feel seen and heard (Rogers, 1961). Essentially, the individual needs to feel psychologically safe. In such conditions, no mental energy is devoted toward self-protection, allowing the energy to be allocated toward one’s own growth process.

### Congruence: Openness to Experience and Learning to Trust the Self

When embedded in a *psychologically safe atmosphere*, an individual would feel enough trust to gradually dare to open themselves up toward their self, listening to the inner processes and becoming more self-aware, particularly of one’s emotional and cognitive movement. This process is ultimately vulnerable, making trust essential. Such openness to one’s experience and listening to the self can lead to a greater understanding of the self and one’s inner communication (Rogers, 1961). Here, the embodied nature of emotions becomes relevant: through this open communication of emotions to the self, individuals can learn to make clear differentiations of their emotions, such that they are able to seek a precise referent to describe that emotion (Rogers, 1961). This differentiation can gradually form into a trust of one’s emotional reactions and a sense of greater understanding of the self. The person can gradually approach a state of congruence between their actual experience and their awareness thereof, as well as exhibit greater outward behavior expressing this congruence (Rogers, 1961).

What this trust additionally entails is a greater acceptance of all the various emotions and reactions that one exhibits. Particularly, it involves accepting those negative states, unfavorable views, fears, and worries as the genuine and authentic representation of one’s organismic experiencing (Rogers, 1961). This is an important aspect of trusting the self: that is, that even difficult emotions are our “friends,” signaling important ideas, and can be dealt without fear.

### Congruence: Autonomy, Responsibility, and Becoming Who You Are

According to Rogers (1961), *an openness and trust of the self* can arise prior to or at the same time as the ability to set the locus of control within the self and become autonomous with one’s decisions and directions in life. Additionally, in setting the locus of control within the self, the individual realizes that they have personal responsibility over their own well-being (Rogers, 1959, 1961). This creates a stronger sense of autonomy, or the belief that one is not a passive victim of circumstances but rather an active force. This stage is therefore one of empowerment of the individual as an autonomous responsible agent (Rogers, 1961). The direction one takes is therefore more internally driven and can express the self more. These changes occur as “moments of movement,” or inner realizations during which something represented in the mind becomes connected with the organic experiencing of the body, and a process of insight occurs when an organic realization becomes a part of the person’s way of representing the self and things in the world.

### Ability to Be a Process, Not a Product: Motivation to Grow Along With Life

When one *feels open to their experiencing, has a good understanding of oneself, feels more autonomous in one’s pursuits and values*, and *trusts in the self*, one learns to be more open to life’s fluctuations without needing instant closure (Rogers, 1961). This means that one can openly find harmony within the fluctuations of life, accepting oneself as a process of becoming rather than a finished product (Rogers, 1961). Furthermore, one embraces change in a vulnerable way rather than seeking safety and closure in rigid patterns of categorization (Rogers, 1961). The security once obtained from a secure social environment becomes, at this point, an inner security, which can serve as a source of courage to face life with all its ups and downs.

### The Prosocial Dimension: Compassion for the Self and Others

Ultimately, this *process of connection and acceptance of the self and one’s emotions* generates an openness toward other people, which manifests as prosociality and greater acceptance of others (Rogers, 1961, 1964). This occurs because the individual no longer fears external threats as much and no longer feels a need to act defensive toward threats to their self-worth, since this worth has become an inner source of security. Such security in turn allows the individual to more readily approach and connect with others with compassion. Rogers (1964) suggested that a person in touch with their organismic growth process can more readily and heartily wish for the well-being of others and the whole world. Accordingly, Rogers’s (1964) view of the individual is based on a positive notion, namely, that a person with high well-being and a strong connection with their growth process values the well-being and growth process of others.

### The Fully Functioning Person

The *process of openness, self-connection, authenticity*, and *vulnerability* ultimately leads to greater well-being for the individual. Upon realizing that the *locus of control* resides within the self, the individual *perceives the personal responsibility that they have over their own well-being*. A person who is in touch with their organismic valuing process is congruent (and feels authentic) and has high levels of unconditional positive self-regard, openness to experience, and compassion and can be considered fully functioning (Rogers, 1961), that is, in a state of optimal psychological and social functioning.

To summarize, the organismic growth process is a theory of necessary change (following the Aristotelian philosophy of necessary and accidental change; see Overton, 1984) whereby the organism naturally matures through a number of psychological stages toward greater well-being. However, although this change is “necessary,” its rate is largely dependent on various social conditions that nourish growth (see Rogers, 1961, 1964). Therefore, the theory is also embedded fundamentally within a social context and is not merely individualistic (Rogers, 1959, 1961, 1964). Furthermore, the theory is based on the concept of holism or the notion that the change process is not solely a process of mechanistic growth of additive positive effects; rather, the stages described above interact and do not progress sequentially (see Rogers, 1961). This means that the theory does not lend itself to clear developmental stages but postulates the existence of necessary mental shifts, or moments of movement, that ultimately lead toward the goal of becoming fully functioning.

## The Fully Functioning Person and Other Humanistic-Existential Theories

Other humanistic-existential theories of the “fully functioning” state rely on the concept of self-transcendence. The existential and humanistic psychologists Frankl and Maslow refer to self-transcendence as either the ultimate need (Maslow, 1971) or as the “essence of existence” (Frankl, 1966, p. 104). Self-transcendence can be defined as the transcendence of one’s ego, accepting the natural world, and moving beyond dichotomies such as us vs. them (see Maslow, 1971; Kaufman, 2020). Kaufman (2020) provides a more recent definition of self-transcendence: “Healthy transcendence is an emergent phenomenon resulting from the harmonious integration of one’s whole self in the service of cultivating the good society” (Kaufman, 2020, p. 217). Healthy transcendence means being in harmony with the rest of humanity similarly than being fully functioning. Moreover, according to Wong (2016), self-transcendence is the act of becoming selfless and responsible over the welfare of others and the world, rather than self-involved. One of the main criticisms of Rogerian theory is that it is too individualistic; that is, it focuses too much on individual’s self-observation rather than on community or social factors (see O’Dwyer, 2012). Therefore, at first glance, self-transcendence seems to offer quite a contrast to the self-observing- and self-awareness-promoting tendencies of growth suggested by the organismic valuing theory. However, a closer look suggests that the organismic valuing process also supports self-transcendence as the ultimate goal of growth. In the organismic valuing theory, becoming fully functioning is in essence a self-transcendent state—the individual becomes more compassionate and more concerned with the well-being of all life forms and the world, as well as takes responsibility their lives and other beings (Rogers, 1959, 1961, 1964). Therefore, the Rogerian theory also considers the social environment crucial for the growth process (accepting and genuine social environment, basic conditions) and that growth entails becoming more compassionate and concerned with the well-being of others and the world; thus, social factors are necessary preconditions and the outcomes of growth. Therefore, the organismic valuing theory could be seen as a theory of growth toward self-transcendence.

## Part 2: Positive Psychological Research Supporting the Organismic Valuing Theory of Growth

To further explore the OVP theory and connect it to current as well as earlier research within positive psychology, we discuss the main assertions of OVP theory described above in light of the current research.

### The Actualizing Tendency

The first concept from OVP is that people are naturally motivated to self-actualize (i.e., to move toward greater well-being). A positive psychological theory called *self-determination theory* (SDT; Deci and Ryan, 2000) postulates a similar idea: given conditions that satisfy one’s basic psychological needs (i.e., relatedness, competence, and autonomy), one will move toward greater self-connection and authenticity (called “intrinsic pursuits” in SDT, which parallel the concepts of “congruence” and “authenticity” in OVP). Joseph (2015) similarly discusses how SDT is based on similar theoretical assumptions of well-being as OVP. These assumptions include that the organism is geared toward growth in an active manner, the organism has the proactive ability to master their internal and external forces (i.e., not be deterministic), and that this growth process can occur when the social conditions for it are favorable. SDT, then, can be seen as directly supporting the metatheory of OVP (Ryan, 1995; Joseph and Murphy, 2013; Joseph, 2015).

The SDT combines an integrative organismic approach to human growth with a social-psychological perspective, whereby it is emphasized that people are influenced not solely by their internal organismic tendencies but also by the social context in which they are embedded. The concept of intrinsically motivated behavior—that is, doing things for pleasure or interest, and not external reward or persuasion—can be considered a behavioral operationalization of the actualizing tendency (Ryan, 1995). Ryan (1995) discusses how intrinsically motivated behaviors support this actualizing tendency (or organismic growth tendency), so long as the social environment is supportive of this and provides the individual with the basic psychological needs of autonomy, competence, and relatedness. In line with OVP theory, SDT suggests that the more intrinsic the behavior is, the more integrated it is with the self. In other words, the more autonomously motivated a person is, the less he or she is heteronomously, or extrinsically, motivated (Ryan, 1995; Deci and Ryan, 2000).

The SDT has ample support as a process of enhancing well-being. The satisfaction of basic needs has been shown to predict well-being in the context of sports (e.g., Ryan and Deci, 2001), school (as teachers’ tendency to give autonomy support; e.g., Jang et al., 2016; Oga-Baldwin et al., 2017), work and organizations (e.g., Deci et al., 2017), and close relationships (e.g., La Guardia and Patrick, 2008), among others. A key point of SDT is how it distinguishes between different types of values (intrinsic and extrinsic values). Having goal aspirations based on intrinsic values is known to be predictive of higher well-being and positive affect, and lower levels of depression and anxiety, compared to aspirations based on extrinsic values (e.g., Kasser, 2002; Niemiec et al., 2009). Similarly, attaining intrinsic goals predicted higher well-being and greater ego-integrity in older adults (e.g., Van Hiel and Vansteenkiste, 2009). Sheldon et al. (2010) also found that having recently progressed in intrinsic goals predicted greater well-being. Overall, intrinsic values are clearly growth enhancing and support the OVP (Ryan, 1995; Deci and Ryan, 2000).

At times, however, people might not seem to be striving toward greater well-being (i.e., do not appear to exhibit the actualizing tendency), instead engaging in behaviors or habits that harm their well-being. Such tendencies, according to Rogers (1961), do not disprove the existence of the growth motivation—instead, they are symptomatic of problems in people’s ability to engage with their OVP due to conditions of worth. The impact of conditions of worth are evident in the study by Harter et al. (1996), who found that adolescents reported suppressing their true selves and altering their behavior to obtain approval when the support they received from their parents was conditional—in other words, they altered their behavior due to perceived conditions of worth. Likewise Kasser et al. (1995) studied young people’s value orientations and found that less nurturing mothering was associated with lower intrinsic (i.e., self-enhancing) goal aspirations and greater extrinsic (i.e., externally placed) goal aspirations, suggesting that conditions of worth relate to remoteness with one’s own OVP and a tendency to live according to extrinsic demands. In fact, many approaches to mental health consider that ill-formed self-concepts or belief systems create a self-fulfilling prophecy to undermine well-being; for instance, correcting such ill-formed thoughts is the goal of cognitive–behavioral therapy (Sanders and Wills, 2012). When the social environment has robbed an individual of their psychological safety, they can easily turn toward chaos and disorganization, such as in the case of insecurely attached children (see Siegel, 2001; Rees, 2008; we also describe attachment in greater detail in the next section).

### Unconditional Positive Regard, Self-Acceptance, and Conditions of Worth

As discussed above, relatedness and connection are a fundamental psychological need (Siegel, 2001; Deci and Ryan, 2000; Brown, 2006, 2012; Fredrickson, 2013). However, the type of connection matters, as they can be either growth enhancing (unconditional positive regard) or growth stifling (conditional positive regard; see Joseph, 2015).

Conditional positive regard refers to valuing another person only when they fulfill a certain condition of worth, for example, showing affection toward one’s child only when they have received high school grades (see Joseph, 2015). Conditions of worth, when internalized, are the birthplace of psychopathology due to their tendency to suppress a natural growth process and one’s ability to be in touch with their authentic experiencing without shame.

Attachment theory illustrates quite well how conditional and unconditional positive regard can influence infants’ attachment styles with their caregivers. Attuned and consistent communication patterns between the caregiver and infant predict a secure attachment style. Secure attachment refers to the ability of the infant to trust that their communication is attended to, and their needs are met with care. Securely attached infants and caregivers show synchrony and attuned communication and consistently reflect (or mirror) one another’s expressions (see Siegel, 2001). Such communication patterns and touch encourage oxytocin release in both parents and infants (Feldman, 2012). Ainsworth et al. (1978) described the behavior of the securely attached infant as one of trust. They can courageously explore their surroundings, using the caregiver as a secure base to return to for comfort and safety. This is much like the security described by Rogers (1959, 1961) in the therapy sessions: creating a space of attunement, unconditional care, and psychological safety in which the client can develop the courage to start to explore their inner world. With psychological safety (i.e., unconditional positive regard), one can release and let go of a need to defend or protect the self against potential dangers (and thereby stay closed up) and thereby grow in courage to tune in with themselves and others.

Insecure attachment patterns, on the other hand, refer to unreliable patterns of care, positive resonance, and attunement (e.g., Rees, 2008). An infant who does not feel that their caregivers have attuned to them forms behavioral patterns of avoidance or anxiety to deal with the insecurity of receiving love (Rees, 2008). Such emotional neglect at an early stage can have numerous negative consequences, including impaired stress regulation ability, lower awareness of one’s bodily signals and emotions, lowered sense of self-worth, distorted views of relationships (e.g., overdependence on others) and the self (e.g., low self-esteem and sense of a lack of independent competence), and a tendency to cling to sameness and security (see Rees, 2008). In light of OVP theory, emotional neglect might lead one to lose touch with their own OVP, leading to a remoteness from the self, potential psychological problems, and rigidity (i.e., clinging to safety and an inability to embrace the insecurities of change).

Siegel (2001) developed the idea of “interpersonal neurobiology,” with emphasis on the fundamental importance of early attachment styles on the child’s ability to form integrated connections in the circuitry of the brain, a coherent sense of self, and good interpersonal relationships. Siegel (2001, 2009) discussed human emotions as a complex system in which each component may come together to function as a unitary system, enabling more adaptive functioning. The opposite of this is the move toward greater rigidity, sameness, and disconnection, which is the basis of mental health problems. In this manner, attuned communication and highly secure relationships are building blocks of greater integration of the system’s components and therefore are fundamental to mental health (see Siegel, 2001, 2009). Siegel (2001) suggested that experiencing positive regard in early communication can enable better self-regulation because it helps build coherence in the person’s neural integration. In this way, interpersonal relationships shape our neural connections, giving rise to our primary sense of self—fundamentally, then, a resonant relationship can aid in the development of a congruent self, much in line with the Rogerian perspective (Siegel, 2001).

Brown (2006, 2012) found in her studies on well-being and shame that at the heart of well-being is the feeling of being worthy of connection with others. She postulates that the ability to reach out to truly connect with another requires vulnerability (i.e., emotional exposure), which in turn requires one to be able to lean into discomfort and have a sense of worthiness in doing so (Brown, 2006, 2012). The opposite of connection is the fear of disconnection—namely, shame. Shame stems from a sense of unworthiness and feeling that one cannot connect with other people. It is a debilitating emotion related to psychological distress (Brown, 2006, 2012). According to Brown (2006, 2012), connection with significant others is the fundamental building block for a good life and mental well-being for the individual, and helps build a sense of worthiness.

On a similar note, Patterson and Joseph (2006) devised a scale for measuring unconditional positive self-regard to assess how therapy can help the individual find a sense of unconditional worthiness and self-acceptance. This relates to the Rogerian perspective in that, through a sensitive connection, individuals can gradually learn to turn the positive regard they receive from the other inwards. Unconditional positive self-regard refers to one’s complete acceptance of the self, with all its complexities, with empathy, openness, and kindness. Patterson and Joseph (2006) found that it was related to higher psychological well-being and happiness. Flanagan et al. (2015) found that people who were higher on unconditional positive self-regard at an initial measurement point had higher levels of posttraumatic growth 3 months later.

Fredrickson (2013) discusses the embodied emotion of love, which she defines as micromoments of positive resonance between two people. This connection is something that Fredrickson describes as a fundamental need, and therefore, its presence and absence can have an important impact on one’s life (Fredrickson, 2013). Love is the ultimate positive emotion, which creates an upwards spiral of positivity and brings well-being. Fredrickson (2013) studied love on a physiological basis and found that it is present in tiny moments of connection, wherein two people attune to each other and release the hormone oxytocin. At the level of brain activity, Stephens et al. (2010) found that when two people truly connect in conversation, their brains show extensive synchrony; this is similar to mirror neuron activity but on a larger level. The synchrony is much lower when the connection between the individuals is not as intense. According to Fredrickson (2013), this love can quite openly occur between any two people and can produce an upward spiral of growth and well-being (Fredrickson, 2013).

### Openness to Experience and Learning to Trust the Self

The concept of learning to trust the self in the Rogerian sense (1961, 1964) involves being more in tune with one’s inner communications, experiencing less fear of listening to and understanding this process, learning to listen to one’s gut feeling, and finding a precise referent for one’s experience (Rogers, 1961). Taken together, the process relates to greater awareness, acceptance, and understanding of one’s inner processing. This can be equated to some degree with Baer et al. (2006) definition of mindfulness, that is, acting with awareness, responding with less reactivity (i.e., pausing before responding or reacting), and being able to find a clear referent (or label) for one’s inner experience (i.e., able to distinguish mental processing), remain non-judgmental (i.e., able to free oneself from the automatic judging of situations), and observe the self with awareness.

Research on mindfulness provides ample evidence that the ability to turn inward, attune to one’s bottom–up (bodily) processing, and pay attention to the present moment without judgment is associated with greater openness to experience (Adair and Fredrickson, 2015). Mindfulness—the degree of awareness and observation of the present moment—is both a state (enhanced in the moment through, for instance, meditative practice) and a trait (ability to be mindful; e.g., Stahl and Goldstein, 2010; Brown et al., 2011). Siegel (2009) discusses how mindfulness helps us reduce our focus on top-down processing (i.e., thinking, planning, analyzing etc.) and turn our attention to our bottom-up bodily reactions in any given moment, which involves utilizing our senses and letting go of the tendency to rationalize, make sense, categorize, or hold to preconceived notions (see Siegel, 2009, 2010). This view resonates closely with the process that Rogers (1961) described for greater personal growth, that is, the person learns to listen more closely to their inner reactions and communication, freeing themselves from their older construals (categorizations and beliefs) that previously guided their beliefs and actions. Consequently, they can open up to each situation and sense each moment as new. Such a person, therefore, does not judge each moment through a prior belief, memory, or category but rather openly embraces the unfolding moment (Rogers, 1961). Therefore, the fully functioning person in the Rogerian sense—who has grown according to their OVP—is likely to be a highly mindful person.

Koole et al. (2009) further suggested that mindfulness could aid in individuals’ finding congruence between implicit (unconscious) and explicit (conscious) self-esteem. Implicit self-esteem refers to one’s unconscious emotional evaluation of the self—if one implicitly feels good about the self and can explicitly describe themselves in a positive way, one’s self-esteem is in congruence and balanced. By contrast, if one implicitly feels bad about the self, but has a positive explicit self-evaluation, they might develop narcissistic tendencies (see Koole et al., 2009). Koole et al. (2009) showed that practicing a short mindfulness intervention led participants to report more congruent self-esteem. This suggests that mindfulness can aid people in connecting with their bodily experience as well as consciously sense this experience. This is an example of what Rogers described as *congruence*, and it has since been found to be associated with high authenticity (see Wood et al., 2008a).

It is also important to mention that Rogerian therapy (1959, 1961) appears to facilitate mindful awareness, which is remarkable given that this construct was not widely known at the time of its development (in the 1950s). The fact that Rogerian therapy sessions were based on a person-centered, non-directive approach—in which the therapist did not lead the client, but rather the client was free to delve into their processing of their own inner information—seems to have induced a state of mindfulness. From a collaboration with Rogers, Eugene Gendlin developed *focusing*—teaching individuals to hold an open and non-judging attention to their “felt sense,” an internal knowing that is directly experienced but has not yet been put into words (Gendlin, 1981), much like the skill of mindful attention. Focusing was later incorporated in emotion-focused therapy (e.g., Greenberg, 2006), in which emotions are seen as fundamental to the construction of self and self-organization; therefore, developing the ability to access them and alter maladaptive emotions is key (Greenberg, 2006). Siegel (2010) also discusses the mindful therapist as being one who is attentively and sensitively present in the moment with the client and capable of attuning in with their client’s inner processing. This sounds very close to the way in which Rogers (1959, 1961) behaved as a therapist, which was based on empathy, genuineness, and being closely attentive and present with the client.

The notion that mindfulness can also be induced through discussion, not merely meditative practice, is also discussed by Langer (1989) and Langer and Moldoveanu (2000). Langer and Moldoveanu (2000) defined mindfulness as the “process of drawing novel distinctions” (p. 1). According to Langer and her colleague, top–down processing (thinking, discussing, etc.) can be mindful if it involves the state of being in the present with an ability to be open, take into account differences in perspectives, and make new distinctions (Langer, 1989; Langer and Moldoveanu, 2000). Langer, therefore, defined mindfulness not as a bottom–up sensory experience but as a cognitive capacity. The Langerian mindfulness construct includes subcomponents of flexibility and the seeking and production of novelty and engagement (Langer and Moldoveanu, 2000). This type of mindfulness—mindfulness through eliciting free thinking of one’s inner processing and associating various aspects of that processing in a novel way—has been also discussed by Saarinen and Lehti (2014). This type of approach could be seen as inducing cognitive mindfulness, which is something that is arguably happening in Rogerian psychotherapy in which the client is processing and drawing greater awareness to their inner processing.

Openness to one’s inner processing was described by Rogers (1961) as a process that is first unfamiliar and frightening, feelings that gradually ebb as they learn to trust the process and lean into the discomfort of such vulnerability. Brown (2012) has discussed the concept of vulnerability as the courage to lean into discomfort and show emotional exposure with courage. According to Brown (2012), the capacity to be vulnerable and let oneself be seen is the birthplace of courage, growth, and well-being, which is much in line with the OVP theory (Rogers, 1961).

### Autonomy, Responsibility, and Becoming Who You Are

A very important part of the OVP theory is that the person learns to place the locus of control within the self and therefore take responsibility over their actions and feel more self-directed (Rogers, 1959, 1961, 1964). Such a tendency requires self-connection, or learning to listen to and recognize the ways in which one construes the world and the self-image, and whether one has been largely governed by influences and ideas coming from extrinsic sources and conditions of worth. Rogers (1961) described this realization as a sense of becoming the subject, instead of the object, of one’s life, referring to the empowering of the personal ability to guide one’s life, rather than feeling like a victim of one’s circumstances.

Santapukki (2006, 2010) discusses the empowerment of the self—that one can and should take responsibility over their lives, that despite the potential support that one can receive, ultimately only they themselves can govern the decisions and changes that can lead to greater personal well-being—as one of the greatest realizations on the path of personal growth. Understanding this responsibility can also be healthy in that one does not rely on codependence for supporting the self but rather can trust the self as the ultimate source of one’s own well-being (Santapukki, 2006, 2010).

Here, the intricate balance between the need for unconditional positive regard from another and the empowering of the autonomous self becomes evident in the OVP theory. According to Rogers (1961), at higher stages of personal growth, when the individual is connecting more with their OVP, the need for unconditional positive regard from another diminishes. The locus of approval turns inwards at this point (i.e., higher unconditional positive self-regard). This is a definitive step toward greater autonomy and embracing of the authentic self.

Wood et al. (2008a) proposed the construct of the authentic personality, based on the Rogers (1959) OVP theory. The dispositional construct of authenticity operationalizes some of the key points of the OVP theory, including the ability to gain autonomy and self-direction instead of behaving according to conditions of worth; understand and listen to one’s inner processing; and gear toward authentic, or intrinsic, behavior. The construct includes three subcomponents: *alienation from the self*, which reflects the state of congruence between true experiencing and conscious awareness of it (i.e., the sense that one is connected to and knows the true self); *authentic living*, or the congruence between conscious awareness of one’s experience and one’s outward behavior of it (i.e., being able to express one’s true self in behavior); and *rejecting external influence*, or one’s ability to be self-governing and reject external pressures and conditions of worth. Very high authenticity therefore reflects a state of congruence between the awareness of one’s real experience and the ability to behave according to that experience in an autonomous way, all the while rejecting external pressure (Wood et al., 2008a). This summarizes some key points of the OVP theory, in which authenticity is a key change taking place in the growth toward becoming fully functioning. Authenticity has been found to be associated with higher well-being, including both subjective and psychological, as well as higher self-esteem (Wood et al., 2008a).

Another conceptually similar way of defining authenticity is given by Goldman and Kernis (2002), who see authenticity as consisting of subcomponents relating to awareness of self (i.e., one’s attributes), an unbiased view of the self, honesty in expression, and openness in relationships. Authenticity has also been defined in different ways, with a greater focus on a sense of identity consistency across social roles (Daukantaitė and Soto, 2014; Robinson et al., 2014), with the premise that a huge variability across social roles might indicate that one puts on a facade (Goldman and Kernis, 2002). Furthermore, Lenton et al. (2013) found that participants tended to feel that calm and low-arousal positive situations and situations reflecting a highly idealistic view of the self were the “most-me” authentic experiences. This suggests that seeing the self favorably, with a focus on strengths, may enhance feelings of authenticity. This is also what Peterson and Seligman (2004) describe as occurring when one identifies their highest character strengths, that is, the strengths feel highly reflective of the authentic self and owned, and motivate behavior. With the varying definitions of authenticity, what seems to be common is the deeply felt sense of being in touch with oneself. Across various studies, such a sense has been shown to be related to higher levels of well-being (Goldman and Kernis, 2002; Lenton et al., 2013; Daukantaitė and Soto, 2014), in line with the OVP. Furthermore, authenticity coupled with positive self-regard might help individuals see the authentic self as reflected through one’s highest potential—top character strengths.

In connection with authenticity and autonomy, self-driven goal pursuits have been shown to be strongly well-being enhancing (Sheldon and Kasser, 1995). Sheldon and Elliot (1999) call such goals *self-concordant*, or consisting of intrinsically placed pursuits that enhance the self and reflect a person’s life-long interest and core values. This theory is likewise based on the OVP in that the self-concordant pursuit can satisfy one’s organismic needs (Sheldon and Kasser, 1995). Participants who were high on self-concordance were also higher on empathy, openness, vitality, and self-actualization (Sheldon and Kasser, 1995).

### Ability to Be a Process, Not a Product: Motivation to Grow Along With Life

According to Rogers (1961), one of the most fundamental mental shifts that a person undergoes as they progress along their OVP is the ability to accept that life, as well as oneself, are in a state of constant flux. The individual must therefore adopt a positive attitude toward growth and development that occurs alongside the changing circumstances of life. This stage is in contrast to a state of rigidity, holding onto set constructs and refusing to change and be open (Rogers, 1961).

This notion of a willingness to embrace change and growth has received ample attention within positive psychology research. For instance, Dweck (2006) coined the terms *fixed* and *growth mindset* (also referred to as entity and incremental beliefs, respectively), which distinguish those that rely on safety and old patterns of behavior and beliefs and those that believe that growth and change are both possible and desirable. The fixed mindset is reminiscent of the Rogerian stage of rigidity mentioned above. People with such a mindset tend to hold implicit beliefs that they possess a determined level of skill and have a determined personality, and those aspects are fixed (Dweck, 2006). This belief can have profound effects on motivation, such that the person develops a strong desire to prove themselves or their level of ability, and fear that they fail to live up to that perceived level (Dweck, 2006). Such a person may be sensitive to failure and feel that failure is deterministic of one’s worth. The growth mindset, on the other hand, is related to a belief in the malleability of one’s traits and to a motivation toward growth through greater effort (Dweck, 2006). Someone with a growth mindset might not be as sensitive to failure, since they do not believe that failures disprove one’s worth but rather indicate that one must try harder (Dweck, 2006).

One construct reminiscent of mindset, particularly in terms of goal motivation, is grit (Duckworth et al., 2007). Grit is the perseverant tenacity to passionately strive toward a long-term goal, even over several years and in spite of setbacks (Duckworth et al., 2007; Duckworth, 2016). In other words, grit entails a devoted motivation and strong abilities for self-regulation toward continuing the pursuit of one’s goal. Vainio and Daukantaitė (2016) recently discussed grit as being akin to the growth motivation of the OVP and tested whether grit would reflect either rigidity or a positive growth tendency. They found that grit was highly related to different well-being types, including life satisfaction, psychological well-being, and harmony in life (discussed below), through the mediators of sense of coherence and authenticity. This was in line with the OVP framework and suggests that grit might reflect a particular connection with the self since it entails such devoted goal pursuit (Vainio and Daukantaitė, 2016). Maddi et al. (2013) further discussed grit in relation to hardiness, suggesting that grit might lack the aspect of existential courage, that is, the ability to change course if the changes in one’s circumstances so demand. However, the fact that grit was related to different aspects of well-being, even harmony, suggests that grit does not undermine well-being or reflect rigidity but serves as a motivation for growth from the perspective of the OVP (Vainio and Daukantaitė, 2016). Grit might in fact be a way in which the actualizing tendency is psychologically manifested. This assertion should be further explored.

The concept of *harmony in life* approaches well-being from a secondary-control perspective. It refers to one’s ability to let go of the need to control one’s life and surroundings and find harmony in the fluctuations of life and feeling a sense of connection with the greater world (Kjell, 2011; Kjell et al., 2016). Kjell (2011) discusses that traditional well-being measures, such as subjective and psychological well-being, tend to take a primary-control view, putting the individual in full control of their life. Perhaps, like Rogers also suggests, at a higher stage of the organismic growth process, one can establish greater levels of harmony, including a greater interconnectivity and prosociality in the context of the wider world, along with the ability to accept that change and growth are inherent aspects of life.

Another construct illustrating the well-being-enhancing effects of accepting the fluctuations of life is *posttraumatic growth* (Tedeschi and Calhoun, 2004). According to Frans et al. (2005), most people (upwards of 80%) will face some form of traumatic event in their lifetime. Some of these might be highly disturbing and can shatter one’s assumptions of the world, forcing one to reckon with a disruption of their belief system. Joseph and Linley (2005) formulated the concept of *growth after adversity* by using the OVP framework, basing their theory on the notion that people are inherently motivated to strive toward greater well-being, which suggests an inborn motivation to move toward coping with or even growing from trauma. They suggest that one takes different paths after trauma. According to one path, they might assimilate the information into their assumptive world by holding onto their previous assumptions and thereby returning to the level of functioning prior to the trauma. In this way, the person shows evidence of resilience or being able to bounce back to previous levels of functioning after an adversity. However, such a state might lead to greater fragility, since the assimilation process might entail distraction from or avoidance of trauma-related information or even blaming the trauma on the self so as to be able to preserve one’s view of the world (see Joseph, 2015). Alternatively, individuals might accommodate trauma-related information within one’s assumptive world—in other words, they might let the trauma alter their worldview. This can occur in either a negative way, in which the person might begin to perceive the world as unjust, leading to a sense of helplessness, or more generally lose their belief in the coherence of life. This can result in negative affect and depressive symptoms (Joseph and Linley, 2005; Payne et al., 2007; Joseph, 2015). However, it is important to notice that depressive symptoms are not necessarily a bad sign; in fact, depression after a cancer diagnosis was associated with greater posttraumatic growth at follow-up in breast cancer survivors, suggesting that depression can serve as a catalyst for growth (Romeo et al., 2020). Yet, when one accommodates the information in a positive way, this means that one rebuilds their assumptive world incorporating the trauma information, but being able to find a new meaning or positive angle with this change in one’s life. Such changes might entail greater appreciation of life, a greater sense of gratitude, greater connection with one’s true values, feelings of authenticity, or a deepened sense of connection with other people (Joseph and Linley, 2005, 2006). Posttraumatic growth is a good example of how the ability to embrace change, uncertainty, and the fluctuations of life may bring about greater levels of well-being and a new level of psychological and social functioning.

While the life-altering and belief-shattering effects of traumatic experiences has been shown to, in many cases, lead to further psychological and social gains for the individual, Roepke (2013) has pointed out that growth can also occur after a positive life event. Roepke (2013) based her theorizing both on the broaden-and-build theory of positive emotions (Fredrickson, 2001) and the *inspire and rewire* theory by Haidt (2003). Both these theories suggest that experiencing positive emotions can build toward further positive states, open up the thought–action repertoire and perception, and inspire growth. The growth-enhancing positive emotions are related to the concept of “self-transcendence,” which denotes a sense of meaning such as gratitude, awe, and inspiration (Peterson and Seligman, 2004; Keltner, 2009). All these can create a sense of connecting to something greater than the self and are in fact often also the types of positive changes described in relation to growth following trauma (see Chun and Lee, 2013). Likewise, approaches to personal growth motivation have focused on the well-being-enhancing effects of a willingness to change and develop in meaningful directions. However, as noted above, accepting such changeability requires one to lean into discomfort as well as be able to face challenges and negative emotions (e.g., anxiety and worry).

Another important concept related to personal growth is eudaimonic growth, which was proposed by Bauer and McAdams (2010). They recognized that people tend to report either safety goals or growth goals as major life goals. Safety goals relate to the protection of the self and staying in one’s safety zone, and are closely related to the rigidity described in the OVP theory. By contrast, growth goals tend to include themes of greater intellectual exploration, understanding, new perspectives, deeper interpersonal connections, or greater enjoyment of life (Bauer and McAdams, 2010). Similarly, growth-related life narratives from older adults appear to be related to meaning-based self-identities and well-being, suggesting that seeing growth and development in one’s life might have positive effects and deliver a sense of meaning (Bauer and Park, 2010).

### Prosocial Dimension: Compassion for the Self and Others

Rogers (1961, 1964, 1980) believed that a person in touch with their OVP—who is growing toward greater functioning and well-being—will be more willing to be prosocial and wish for the well-being of others. In this sense, Rogers (1961, 1964) emphasized that the quest for one’s own well-being is ultimately prosocial. His view on the depth of this direction is effectively captured in the following passages:

“I find it significant that when individuals are prized as persons, the values they select do not run the full gamut of possibilities. I do not find, in such a climate of freedom, that one person comes to value fraud and murder and thievery, while another values a life of self-sacrifice, and another values only money. Instead there seems to be a deep and underlying thread of commonality. I believe that when the human being is inwardly free to choose whatever he deeply values, he tends to value those objects, experiences, and goals which make for his own survival, growth, and development, and for the survival and development of others. I hypothesize that it is *characteristic* of the human organism to prefer such actualizing and socialized goals when he is exposed to a growth promoting climate” (Rogers, 1964, p. 166).

“The psychologically mature person as I have described him has, I believe, the qualities which would cause him to value those experiences which would make for the survival and enhancement of the human race” (Rogers, 1964, p. 167).

In other words, Rogers (1964) believed that the ultimate stage of development within the OVP comes after the person realizes one’s responsibility toward oneself, and has gained a sense of the interconnectedness of the world and place within it. In some ways, it seems that the unconditional positive regard and acceptance of Rogerian theory are first given to the individual from another to enhance the favorable conditions of growth, after which this regard is given to the self and, ultimately, toward others and the world. This process resonates very closely with the concept of loving–kindness meditation, wherein one learns to cultivate compassion and kindness first toward the self, then toward close others. These feelings are soon extended to increasingly distant others, finally reaching the world at large (Hoffmann et al., 2011).

Hoffmann et al. (2011) discussed loving–kindness meditation and its effects on promoting compassion. Fredrickson et al. (2008) discussed loving–kindness meditation in relation to the broaden-and-build theory of positive emotions, which suggests that positive emotionality builds on itself and creates growth in positivity, such that one’s inner well-being radiates outside and helps build better social bonds (Fredrickson, 2001). Loving–kindness meditation can enhance a variety of positive emotions along with the various aspects of eudemonic well-being such as environmental mastery, positive relations, mindfulness, and reductions in depressive symptoms (Fredrickson et al., 2008). In addition, loving–kindness can enhance feelings of altruism (Wallmark et al., 2012), suggesting that this exercise might enable a broader focus for compassion and foster feelings of connection to the wider world (Fredrickson et al., 2008). Cultivating compassion, therefore, has multiple benefits, and its role in mental health and resilience seems robust (MacBeth and Gumley, 2012). In these ways, the broaden-and-build theory and the cultivation of compassion support the Rogerian OVP theory.

Resonating quite closely with Rogers (1961, 1964) concepts, Van Hiel et al. (2010) study found that extrinsic pursuits for financial success and materialism were related to higher levels of prejudice. When one does not strive for goals that enhance one’s own OVP, there is a greater tendency to categorize and be less open to others. By contrast, the intrinsic pursuit of community concern was inversely related to prejudice and positively related to prosocial tendencies and concern over other’s well-being (Van Hiel et al., 2010). Extrinsic pursuits are also related to lower self-connection or self-alienation, which is considered to reflect the less-than-optimal developmental trajectories and is associated with a loss of connection to one’s growth motivation (see Sheldon and McGregor, 2000; Kasser et al., 1995), greater materialistic ideation (Froh et al., 2011), and greater psychological distress (Kasser, 2002). Intrinsic pursuits, on the other hand, are related to a greater striving toward well-being and a more open attitude toward others and the well-being of the world at large. These are together in line with the Rogers (1961, 1964) notion that engaging in one’s own growth process helps make one more willing to endorse the growth of others.

A rather similar construct to the prosocial tendencies resulting from the growth process is gratitude as a general orientation to life, as defined by Wood and colleagues (Wood et al., 2008b, 2010). Having a grateful orientation is associated with a generally greater appreciation of one’s life and other people, as well as greater experiences of awe when experiencing something beautiful, a greater sense of the “fragility of life,” and greater tendency to behave in ways that express gratitude. Such an orientation is associated with a greater sense of interconnectivity between the self and the surrounding world (Wood et al., 2010) and a higher willingness to give back to society (see Froh et al., 2011). Furthermore, gratitude is often reported as a benefit that occurs with growth following trauma (Chun and Lee, 2013).

### The Fully Functioning Person

The positive psychological construct of optimal well-being—that is, the concept of *flourishing*—resonates with the Rogers (1959, 1961) fully functioning person. For Seligman (2012), flourishing is the possession of both high hedonic (positive emotion) and high eudaimonic (well-being capacities) functioning. According to his theory of well-being, the constructs of positive emotion, engagement, relationships, meaning, and accomplishment (PERMA) are regarded as goals that individuals strive toward for intrinsic reasons (Seligman, 2012). All of these concepts have correlates in Rogers (1961). Positive emotions are a by-product of the well-being growth process, while engagement is considered a reflection of a connection with the self accompanied by intrinsically motivated directions and autonomy. Relationships are emphasized in both enabling a psychologically safe atmosphere for the growth to others, as well as having greater sense of interconnectedness and compassion. Meaning is enhanced by a self-driven and authentic attitude toward one’s endeavors as well as a sense of interconnectedness to humanity and the world.

Keyes (2002) theory of flourishing defines the person with optimal levels of mental health as having high subjective well-being (life satisfaction and positive emotion; Diener, 1994), high psychological well-being (autonomy, environmental mastery, personal growth, purpose, positive relationships, and self-acceptance; Ryff, 1989), and high social well-being (social contribution, coherence, integration, actualization, and acceptance; Keyes, 1998). Keyes (1998) social well-being includes the sense that the social world is sensible and coherent and, through a sense of belonging, can help one grow and actualize. While the Rogerian theory does not as specifically define the various dimensions of social well-being, it does expound on the role of the social environment in satisfying the basic psychological needs of feeling accepted and supported in one’s growth. Likewise, it clarifies the process by which individuals grow in their subjective, psychological, and social well-being. In sum, the Rogerian OVP theory seems to illustrate the process of becoming a flourishing individual.

## Part 3: Some Implications From the Rogerian Organismic Valuing Theory for Positive Psychology and Intervention

Understanding personal growth as a process that unfolds in individuals through certain crucial mental shifts could help immensely in both better understanding well-being theoretically, as well as how to promote well-being in applied settings, such as in therapy and schools. This integration can have implications for both theory and practice.

### Theoretical Implications

Adopting a theory of growth grounded in the OVP theory would have several important theoretical implications. First, the OVP is a theory of the process of personal growth and well-being, which would be a fruitful basis for further empirical explorations of this process. Personal growth has not yet been discussed comprehensively within positive psychology nor studied empirically, since it is a complex unfolding process requiring a holistic dynamic paradigm where the individual is regarded as a complex system that functions and develops as a totality through the convergence of subsystems at different levels (Magnusson, 1985, 1998). This person-oriented approach, which stems from the interactionist research paradigm, focuses on studying and interpreting information about each subsystem in relation to the whole (Bergman and Magnusson, 1997), even though a degree of decontextualization and simplification of the systems under scrutiny are unavoidable (Nilsson, 2015). Thus, studying individuals in terms of profiles of individual characteristics relevant for the growth and well-being over time by using person-oriented methods would provide an in-depth understanding of the whole person.

Second, the OVP considers well-being as an unfolding process characterized by constant change and reinvention—while there are tentative personal growth stages, it fundamentally suggests that the well-being process is ongoing and open. Accordingly, a growing individual is simultaneously growing in terms of inner strength and in a constant state of vulnerability to the ebbs and flows of life, which can readily change their course. Engaging with the authentic growth process can help the individual openly and acceptingly face and overcome these dips in the growth process. Personal growth, in this way, can be thought of as a process of befriending the self and thereby gaining confidence to face life’s ups and downs.

Third, a theory of personal growth based on the OVP would be embedded in a view of the person as being an active agent motivated to grow, become more complex, more closely connect with and express their authentic self, and better fulfill their own potential. In other words, the theory sees the person as proactive. This proactivity is the default of the individual unless this growth process is stifled by the environment. This idea is somewhat radical in that the underlying assumption is that the person has an internal drive for well-being and growth that is inherently proactive and *prosocial.*

Fourth, the theory would suggest that the psychosocial environment is exceedingly important for nurturing (or stifling) the growth of this agentic person. More specifically, the natural organic growth process is either nourished by an environment that offers the fulfillment of their basic psychological needs or stifled by a lack of these needs (Rogers, 1961, 1980). Furthermore, each relationship can be defined as growth enhancing or stifling, which would call for a common social responsibility. While an individual has an inner drive for growth, complexity, and autonomy, and is capable of attaining this, the value system of the social environment can still alienate the individual from their growth process. Therefore, the social environment must be regarded as fundamental to adopting a growth mindset, well-being, and compassion. In sum, the organismic valuing theory could be seen as a metatheory for positive psychology, in that it is a holistic theory of well-being as a process that can incorporate a vast amount of positive psychological constructs and theories within it.

### Practical Implications

Rogerian growth theory can also inform therapeutic and educational interventions aimed at promoting the personal growth process. First, in order to enhance the growth process, the basic psychological conditions of unconditional care, authenticity, and acceptance must be present in order to override any defensive responses. The use of positive psychology in clinical psychology settings could elicit the growth process by helping individuals deal with trauma and promoting their positive potentialities, since either one alone would contribute an incomplete picture of the individual’s situation and growth potential (see Wood and Tarrier, 2010). In an atmosphere of genuineness and acceptance, individuals can more vulnerably engage with the process of growth (see Rogers, 1961, 1959). As discussed by Wilmots and colleagues (2020), a positive therapeutic relationship is essential for the alleviation of problems such as depression. In a school context, the satisfaction of basic psychological needs, which would ensure a psychologically safe school atmosphere through an autonomy-supportive teaching style (see Núnez and León, 2015), is related to student motivation and well-being (Núnez and León, 2015; Liu et al., 2017) and might provide a growth-promoting atmosphere in the classroom.

Positive psychology therapeutic interventions can be useful for the elicitation of the growth process. The actualizing tendency is something that the individual needs to gain a connection to themselves (i.e., to be able to attune to their inner communications). Mindfulness, both in the form of mindfulness meditation and cognitive mindfulness through open conversation (see Langer, 1989; Langer and Moldoveanu, 2000), can be an effective way of doing this. Through mindfulness, one can learn to connect to the bottom–up processing of the body and mind and focus on the present moment (Adair and Fredrickson, 2015). In cognitive mindfulness, one may learn to better understand one’s cognitive processes and gain insight into the ways in which one represents reality and the self (see Langer and Moldoveanu, 2000; Saarinen and Lehti, 2014). Furthermore, since mindfulness may help one in attaining balance between explicit and implicit self-esteem (Koole et al., 2009), it may help in, for instance, mitigating narcissistic tendencies or gaining better self-acceptance.

Another way to engage with the actualizing tendency might be through exercises encouraging self-reflection on one’s values. Such exercises might help one to gain a greater connection with the self and a better understanding of one’s sources of intrinsic motivation. Promoting a self-connection may in turn help individuals understand themselves and set more self-concordant goals (see Sheldon and Elliot, 1999), which in turn will elicit intrinsic motivation and promote the gritty pursuit of such goals (as grit is related to authenticity: see Vainio and Daukantaitė, 2016), thereby enhancing the growth process.

Interventions that promote meaning-making of difficult experiences such as trauma or illness can enhance clients’ sense of meaningfulness and well-being (i.e., self-efficacy, optimism, and self-esteem as in Lee et al., 2006). For instance, in the logotherapeutic tradition, it is recognized that clients must make sense of their experiences and recognize the greater meaning behind their suffering, which can elicit further growth and a sense of transcending of the suffering (Frankl, 1969).

Higher unconditional self-regard could be promoted through the strengths approach provided by positive psychology. Positive psychological interventions focus on promoting well-being through attention to individuals’ strengths rather than their weaknesses, such as learning to recognizing one’s strengths and considering how to use them more in everyday life (e.g., Park and Peterson, 2009; Seligman et al., 2009). Basing goal pursuits on strengths may enhance motivation and feelings of authenticity more than basing goals on improving weakness (Seligman et al., 2009; Seligman, 2012). Higher levels of both unconditional positive self-regard and compassion can be rehearsed by self-compassion exercises (see Neff, 2011) or loving–kindness meditation (Fredrickson et al., 2008).

Furthermore, understanding personal growth as a process has benefits for education, particularly positive education, which aims to integrate scientific understanding of well-being in school curricula to help children gain greater capacities for well-being (see Seligman et al., 2005; Seligman et al., 2009; Norrish et al., 2013; Norrish, 2015). The OVP theory could help in identifying those core conditions that might help young people gain greater well-being capacities. Furthermore, it would allow students to be viewed more as active agents willing to pursue their greatest potential when the circumstances for this—including the satisfaction of their basic psychological needs—are suitable. Seeing children in this light might help to contextualize problem behavior or a lack of interest and attention, thereby identifying them as symptoms of the students’ basic psychological needs going unfulfilled.

It is important to note that Rogers (1959, 1961) believed that conditions of worth—conditions placed from the outside on individuals’ worthiness as a person—are the source of psychopathological symptoms. Therefore, in order to follow the Rogerian therapeutic tradition and enhance the OVP, such conditions need to be avoided in all interventions—that would mean being very careful about “prescribing” positive psychology to clients without consideration of their sensitivities and personalities. The positive should never become a condition of worth for the individuals during a treatment or intervention. Even using character strengths interventions should be done with utmost sensitivity to avoid imposing strengths on individuals that may feel inauthentic or a condition of worth. Instead, interventions should be centered on authenticity, autonomy, and description over prescription (see also Linley et al., 2006). Moreover, positive psychology interventions should not become evaluations of student performance—since such evaluations would become evaluations of personality rather than evaluations of performance, which should never be the case in education (see Keltikangas-Järvinen, 2016, for the importance of evaluating performance and never personality or temperament).

In general, it is advisable for therapeutic and educational relationships to focus on promoting the personal growth process, basing the efforts on building eudaimonic well-being capacities over time in order to help the individual engage with their organismic valuing process in a psychologically safe social environment, avoiding conditions of worth. Such efforts could prove beneficial for the long-term growth of individuals.

### Limitations of the Present Review

Due to space limitations, this present review lacks a more comprehensive look at the controversy between positive and humanistic psychology, which is discussed elsewhere (see Friedman, 2008; Waterman, 2013). Furthermore, a more thorough exploration of the differences and similarities between the OVP theory and other humanistic-existential theories was beyond the scope of this review. Such explorations would help to highlight more fruitful areas of empirical investigation. The common criticisms toward Rogerian theory were likewise not discussed in this review, although they can be found elsewhere, such as in Friedman (2008). Generally, Rogers has been criticized for his highly individualistic theories and naively positive view of the person (Friedman, 2008). However, such individualism is arguably present throughout every field of psychology, including positive psychology (see Christopher, 1999); as such, it is not solely a problem of humanistic theories. Even so, addressing this criticism is necessary for ensuring wider applicability of Rogerian theory. We believe that the Rogerian fully functioning person is a more collectivist state in which the individual is particularly concerned for the welfare of other beings and in harmony with the world.

## Conclusion

The Rogerian OVP theory provides a holistic view of the personal growth process across life and can be used to integrate the often disparate pieces of knowledge within well-being research under a coherent metatheory. The call for integration is based on a call for greater cowork between the subfields of positive and humanistic psychology, utilizing the strengths inherent in each subfield. Both can serve our understanding of growth: indeed, we suggest that the best way of understanding growth is through a process of differentiation (i.e., getting more detailed knowledge of the various relevant psychological variables involved in the process), organization (i.e., organizing the various separate variables into theories), and integration (i.e., integrating the variables and subtheories to an overarching metatheory that can better explain the interconnection of the various elements). Along these lines, positive psychology acts as both the differentiation and organization stages, but some humanistic theories, particularly the organismic valuing theory, could serve as an integrative metatheory, providing a holistic view. Beyond this more individual level of inquiry, a call for the integration of different fields of science could add to our understanding growth in various contexts.

## Author Contributions

MM has contributed the main body of text and the main ideas. DD has contributed to the construction of the text and refinement of ideas and provided extensive feedback and commentary. Both authors contributed to the article and approved the submitted version.

## Conflict of Interest

The authors declare that the research was conducted in the absence of any commercial or financial relationships that could be construed as a potential conflict of interest.
