# Corrigendum: Revisiting the Organismic Valuing Process Theory of Personal Growth: A Theoretical Review of Rogers and Its Connection to Positive Psychology

**DOI:** 10.3389/fpsyg.2021.698829

**Published:** 2021-06-03

**Authors:** Mia M. Maurer, Daiva Daukantaitė

**Affiliations:** Department of Psychology, Lund University, Lund, Sweden

**Keywords:** organismic valuing process, personal growth, integration, humanistic psychology, positive psychology, well-being

In the original article, there was an error. Two places where Rogers is directly quoted, the citation marks around the quotation have been omitted in the published article.

A correction has been made to *Part 2: Positive Psychological Research Supporting the Organismic Valuing Theory of Growth*, subsection *Prosocial dimension: Compassion for the self and others*.

“I find it significant that when individuals are prized as persons, the values they select do not run the full gamut of possibilities. I do not find, in such a climate of freedom, that one person comes to value fraud and murder and thievery, while another values a life of self-sacrifice, and another values only money. Instead there seems to be a deep and underlying thread of commonality. I believe that when the human being is inwardly free to choose whatever he deeply values, he tends to value those objects, experiences, and goals which make for his own survival, growth, and development, and for the survival and development of others. I hypothesize that it is *characteristic* of the human organism to prefer such actualizing and socialized goals when he is exposed to a growth promoting climate.” (Rogers, 1964, p. 166).

“The psychologically mature person as I have described him has, I believe, the qualities which would cause him to value those experiences which would make for the survival and enhancement of the human race.” (Rogers, 1964, p. 167).

The authors apologize for this error and state that this does not change the scientific conclusions of the article in any way. The original article has been updated.
